# Editorial: Exploring motor imagery across the lifespan – gaps between practical applications and theoretical frameworks

**DOI:** 10.3389/fpsyg.2025.1696821

**Published:** 2025-10-03

**Authors:** Pedro Duarte-Mendes, Maurizio Bertollo, André Ramalho, Daniel Almeida Marinho

**Affiliations:** ^1^Department of Sports and Well-Being, Polytechnic University of Castelo Branco, Castelo Branco, Portugal; ^2^Sport Physical Activity and Health Research & Innovation Center (SPRINT), Castelo Branco, Portugal; ^3^BIND-Behavioral Imaging and Neural Dynamics Center, Department of Medicine and Aging Sciences, University G. d'Annunzio of Chieti-Pescara, Chieti, Italy; ^4^PSYLAB – Faculty of Human Kinetics, University of Lisbon, Lisbon, Portugal; ^5^Department of Sport Sciences, University of Beira Interior, Covilhã, Portugal; ^6^Research Center in Sports Sciences, Health Sciences and Human Development (CIDESD), Covilhã, Portugal

**Keywords:** imagery, motor control, neuroplasticity, learning, mental practice, mental skills, action representation

Motor imagery (MI), understood as the multisensory mental simulation of action, engages motor planning and higher-order cognitive networks, linking motor control with broader cognitive functions. Beyond simple rehearsal, MI reorganizes neural activity and supports the acquisition, refinement, and retention of motor skills throughout the lifespan. Its benefits have been demonstrated across rehabilitation, education, music, and sport (Bach et al., 2024; Hurst and Boe, 2022). When tailored to individual needs, MI protocols closely mirror physical training, serving as tools for optimization, adaptation, and recovery (Mendes et al., 2016). Yet despite its wide-ranging applications, key questions remain about how the quality of MI should be assessed, why its effectiveness varies across individuals and tasks, and how complex laboratory findings can be meaningfully translated into real-world contexts.

This Research Topic, *Exploring motor imagery across the lifespan – gaps between practical applications and theoretical frameworks*, addresses these questions through nine complementary contributions. Together they reframe MI as a dynamic, context-sensitive process that requires precise theoretical and methodological treatment.

The opening article by Villabona Orozco et al. examines the neurophysiological foundations of MI by investigating whether mu and alpha oscillations (8–14 Hz) serve as reliable markers of MI success. Electroencephalography recordings were collected from 19 healthy young adults performing the Test of Ability of Movement Imagery, and rhythmic activity was analyzed using parameters derived from the Better Oscillation Detection Method. Contrary to expectations, the study found no consistent associations between oscillatory magnitude and either intra-individual performance or inter-individual ability. Nonetheless, a significant reduction in amplitude during the initiation phase of imagery suggests a transient window of early neural engagement. These findings highlight the variability of sensorimotor rhythms and contribute to ongoing debates regarding their utility as biomarkers in MI research.

Where the previous authors focus on internal signals, Chye et al. explore how action observation combined with MI (AOMI) contributes to early-stage motor learning, focusing on the moderating role of model type. In a structured acquisition protocol involving novices learning the Ankle Pick takedown, they compare the effects of self- vs. other-model AOMI training. While physical practice remains the primary driver of performance gains, differences between model types suggest that the cognitive processing of observed movement varies meaningfully. These results point to the potential value of mixed-model strategies to enhance AOMI-based interventions, particularly in complex skill learning.

The capacity of motor imagery to serve public-health aims is illustrated by Zhou et al., who address the growing challenge of childhood myopia by testing whether MI, integrated into physical education, can serve as a non-pharmacological strategy to support visual and cognitive development. In a 16-week school-based intervention involving 154 children across four groups, they combined MI with visual tasks in various configurations. The results suggest that such integration may stimulate ciliary muscle activity while also enhancing motor and cognitive outcomes. By positioning MI as both a developmental and preventive tool, the study expands its relevance into the domain of public health and offers a model for holistic, school-based interventions.

Technology-mediated MI receives attention in the study by Bedir et al., who extend its scope into immersive environments by evaluating a Virtual Reality-Based Imagery (VRBI) protocol designed to enhance kinesthetic MI and muscle activation in athletes. Compared to established approaches such as Visual Motor Behavior Practice (VMBP) and Video Modeling (VM), the VRBI model demonstrated superior outcomes in both sensory vividness and neuromuscular adaptation. These findings suggest that virtual reality can amplify the effectiveness of mental training by enriching the sensorimotor experience, pointing toward new possibilities for imagery-based interventions in applied sport science.

Cognitive outcomes are the focus of two network meta-analyses. Song et al. examine how physical activity enhances cognitive function in children with attention-deficit/hyperactivity disorder, focusing on working memory as a core area of impairment. Their meta-analysis comparing exercise modalities shows that cognitively engaging aerobic activities yield the strongest benefits. The study offers compelling evidence for targeted, non-pharmacological interventions and highlights the importance of tailoring intensity and structure to optimize cognitive outcomes in clinical and educational settings. Guo et al. contribute to the understanding of youth cognitive development through a systematic review and network meta-analysis comparing the effects of various exercise modalities on working memory in healthy children and adolescents. Their findings reveal domain-specific benefits: dance enhances accuracy, while aerobic activity improves processing speed. These results support targeted physical activity programs aligned with distinct cognitive goals, with clear implications for educational practice and pediatric health promotion.

Motivation represents another critical layer. Viveiros et al., drawing on Self-Determination Theory and its hierarchical model, highlight how autonomy, competence, and relatedness shape adherence, especially in gym-based contexts. Their review links theory and evidence to support motivation-sensitive strategies for promoting sustainable behavior change.

Psychological infrastructure is examined further by Whitty et al. synthesize evidence on psychological profiling in youth sport academies, situating their review within evolving biopsychosocial models of athletic development. Focusing on male athletes in team-based sports, they identify key psychological attributes that shape long-term trajectories. By aligning their findings with the International Classification of Functioning, Disability and Health (ICF) framework, the study offers a practical model for supporting both performance and wellbeing, reinforcing the need for integrated psychological monitoring in elite youth sport settings.

Aging is considered in the meta-analysis by Fierro-Marrero et al., which compares MI abilities in younger and older adults. The review found limited evidence of temporal congruence between imagined and executed movements in older adults, due to wide confidence intervals and study heterogeneity. These findings highlight both the partial preservation and the partial decline of MI abilities with age, depending on task type and measurement dimension.

Collectively, these studies recast MI as a context-sensitive practice shaped by neural, psychological, and social factors. Rather than a fixed technique, MI emerges as a dynamic phenomenon that reflects human variability. Advancing the field will require cross-disciplinary collaboration that connects neuroscience with pedagogy, technological development with clinical insight, and theoretical precision with lived experience. A sustained focus on individual differences and contextual relevance will be essential to ensure both efficacy and ethical integrity.
